# Epigenetics—Beyond the Genome in Alcoholism

**DOI:** 10.35946/arcr.v34.3.04

**Published:** 2012

**Authors:** Bela G. Starkman, Amul J. Sakharkar, Subhash C. Pandey

**Affiliations:** **Bela G. Starkman***is a graduate student in the Departments of Anatomy and Cell Biology and Psychiatry, the University of Illinois, Chicago, Illinois. Dr. Pandey also is a VA career scientist at the Jesse Brown Veterans Affairs Medical Center, Chicago, Illinois.*; **Amul J. Sakharkar, Ph.D.,***is a research assistant professor in the Department of Psychiatry, the University of Illinois, Chicago, Illinois. Dr. Pandey also is a VA career scientist at the Jesse Brown Veterans Affairs Medical Center, Chicago, Illinois.*; **Subhash C. Pandey, Ph.D.,***is a professor and director of Neuroscience Alcoholism Research in the Department of Psychiatry and Department of Anatomy and Cell Biology at the University of Illinois, Chicago, Illinois. Dr. Pandey also is a VA career scientist at the Jesse Brown Veterans Affairs Medical Center, Chicago, Illinois.*

**Keywords:** Alcoholism, alcohol use disorders, genome, epigenome, genetic factors, epigenetics, DNA, brain, amygdala, neuronal circuits, chromatin, histone, DNA methylation, histone deacetylase, histone acetyltranferase, DNA methyltransferase, treatment

## Abstract

Genetic and environmental factors play a role in the development of alcoholism. Whole-genome expression profiling has highlighted the importance of several genes that may contribute to alcohol abuse disorders. In addition, more recent findings have added yet another layer of complexity to the overall molecular mechanisms involved in a predisposition to alcoholism and addiction by demonstrating that processes related to genetic factors that do not manifest as DNA sequence changes (i.e., epigenetic processes) play a role. Both acute and chronic ethanol exposure can alter gene expression levels in specific neuronal circuits that govern the behavioral consequences related to tolerance and dependence. The unremitting cycle of alcohol consumption often includes satiation and self-medication with alcohol, followed by excruciating withdrawal symptoms and the resultant relapse, which reflects both the positive and negative affective states of alcohol addiction. Recent studies have indicated that behavioral changes induced by acute and chronic ethanol exposure may involve chromatin remodeling resulting from covalent histone modifications and DNA methylation in the neuronal circuits involving a brain region called the amygdala. These findings have helped identify enzymes involved in epigenetic mechanisms, such as the histone deacetylase, histone acetyltransferase, and DNA methyltransferase enzymes, as novel therapeutic targets for the development of future pharmacotherapies for the treatment of alcoholism.

Alcohol is one of the most widely used addictive drugs, and continued use and abuse can lead to the development of tolerance and dependence (Koob 2003*a*; Tabakoff et al. 1986). Numerous studies have shown that both genetic and environmental risk factors play a role in the development of alcoholism (Ducci and Goldman 2008; Edenberg and Foroud 2006; Farris et al. 2010). Genetic studies in both humans and animal models of alcoholism (Contet et al. 2011; Crabbe et al. 2006; Pignataro et al. 2009; Spanagel et al. 2010; Tabakoff et al. 2009) have identified several genes that may be critical in the pathophysiology of alcoholism (see figure 1). Recently, researchers have identified mechanisms that result in heritable changes in gene expression but are caused by other processes than changes in the underlying DNA sequence (i.e., epigenetic mechanisms) as a promising area of research to better understand the molecular mechanisms of human diseases, including psychiatric and alcohol use disorders (AUDs) (Moonat et al. 2010; Tsankova et al. 2007). This article reviews some of the epigenetic mechanisms that seem to play a role in the development of AUDs.

## What Is Epigenetics?

The genome encompasses the complete set of genetic material (i.e., DNA) that determines the development of an organism and all its traits and characteristics (i.e., the phenotype). Changes (i.e., mutations) in the DNA can lead to the development of various diseases, including AUDs. In comparison, the epigenome, as first defined by Waddington (1942), refers to chemical modifications that occur within a genome without changing the DNA sequence (Holliday 2006; Murrell et al. 2005; Waddington 1942). Epigenetic alterations include the direct addition of methyl groups to (i.e., methylation of) DNA and the chemical modification of the proteins around which the DNA is wrapped (i.e., histone proteins) to form the chromosomes. Both of these mechanisms work in concert to remodel the structure of the protein–DNA complex (i.e., the chromatin) and regulate gene expression^1^ (Kornberg 1974; Olins and Olins 1974; Hsieh and Gage 2005).

Chromatin is made up of units called nucleosomes, which consist of approximately 147 base pairs of DNA wrapped around a complex of eight histone proteins that comprise the histone core (figure 2) (Jenuwine and Allis 2001; Smith 1991). This core structure, which also is referred to as the histone fold domain, consists of a central heterotetramer of histones H3 and H4 and two heterodimers^2^ of histones H2A and H2B (Luger et al. 1997; Smith 1991). The histone proteins assemble at one end called the carboxy (C) terminal to form the histone core, with the other end, in the amino (*N*)-terminal “tail” region, projecting out from the histone core (figure 2). This *N*-terminal region is made up mainly of the amino acids lysine and arginine (Hsieh and Gage 2005; Mersfelder and Parthun 2006). The majority of epigenetic histone modifications occur at the *N*-terminal tail of the histones and include methylation as well as addition of acetyl groups (i.e., acetylation), phosphate groups (i.e., phosphorylation), binding of a small molecule called ubiquitin (i.e., ubiquitylation), or addition of a molecule called ADP ribose (i.e., ADP-ribosylation) (Jenuwine and Allis 2001; Smith 1991). The most recognized and well established of these epigenetic modifications include histone acetylation and histone and DNA methylation, which control the accessibility of chromatin to essential transcriptional proteins mediating the first step in the conversion of the genetic information encoded in the DNA into mRNA (i.e., transcription), which then serves as template for the synthesis of protein products in a process called translation. By controlling DNA accessibility, these epigenetic modifications play a crucial role in the regulation of gene transcription (Abel and Zukin 2008; Feng and Fan 2009).

Another epigenetic regulatory mechanism involves microRNAs (miRNAs), a class of noncoding RNAs approximately 21 to 23 building blocks (i.e., nucleotides) in length, which are involved in the posttranscriptional modulation of gene expression and function (Bartel 2009). Altered regulation of these specific epigenetic mechanisms plays a pivotal role during the onset of disease (Bonasio et al. 2010). This review discusses the implications of abnormal chromatin remodeling and miRNAs as major factors in several brain disease processes, including AUDs. It also considers the impact of current epigenetic research on the alcohol field and the therapeutically significant function of compounds called histone deacetylase (HDAC) inhibitors in the prevention and treatment of alcoholism.

## Role of Histone Modifications in the Brain During Alcoholism

### Histone Acetylation

Histone modification involving acetylation is regulated by two types of enzymes—histone acetyltransferases (HATs), which add acetyl groups to histones, and HDACs, which remove acetyl groups from histones. Both types of enzymes dynamically interact to regulate the remodeling of the chromatin architecture and gene expression (Abel and Zukin 2008; Hsieh and Gage 2005).

#### Histone Acetyltransferases

Several studies have indicated that regulation of histone acetylation is important during formation of memories in a brain region called the hippocampus (Guan et al. 2009; Levenson et al. 2004). Furthermore, alterations in histone acetylation are associated with age-dependent memory impairment in mice (Peleg et al. 2010). One mechanism through which histone acetylation and thus chromatin remodeling may be regulated involves a protein called cyclic-AMP responsive–element binding (CREB) protein. This protein helps modulate the transcription of certain genes by binding to a specific sequence on the DNA after it has been activated by phosphorylation. Phosphorylated CREB (pCREB) then recruits another transcriptional co-factor called CREB-binding protein (CBP), which contains intrinsic HAT activity. CBP, along with another molecule called p300, enzymatically remodels the nucleosome by transferring acetyl groups to histones, thereby allowing the chromatin to exist in a relaxed conformation that is accessible for transcription (figure 3) (Chrivia et al. 1993; Hsieh and Gage 2005; Liu et al. 2008; Martinez-Balbas et al. 1998).

CBP is essential for both short-term and long-term memory formation and consolidation (Chen et al. 2010; Korzus et al. 2004). A recent study in a mouse model of Alzheimer’s disease expressing abnormal CREB functioning demonstrated that delivery of the CBP gene into the brain increased the levels of a molecule known as brain-derived neurotrophic factor (BDNF), which correlated with improved learning and memory (Caccamo et al. 2010). Of interest, alterations in both memory capacity and responses to stress also have been observed in mice that lack a molecule called p300/CBP-associated factor (PCAF), which has a similar function to CBP and also has HAT activity (Kalkhoven 2004; Maurice et al. 2008).

Correction of deficits in CBP function increasingly has become an important target in the treatment of neurological disorders, including Rubinstein-Taybi syndrome (RSTS), Huntington’s disease, Alzheimer’s disease, and amyotrophic lateral sclerosis (Klevytska et al. 2010; Rouaux et al. 2004; Selvi et al. 2010). Mutations in the CBP gene are prevalent in RSTS (Alarcon et al. 2004; Bartsch et al. 2005; Roelfsema et al. 2005). For example, exon deletions in the genes that encode p300 and CBP have been detected in children with RSTS (Tsai et al. 2011). The changes in CBP function described in these neurological disorders are similar to neuroadaptations observed in alcoholism; therefore, CBP–HAT may be an important factor in the development of alcoholism (see figures 4 and 5).

It is well established that CREB protein functioning is important during long-term memory formation, synaptic plasticity, and addiction (Abel and Zukin 2008; Alberini 2009; Carlezon et al. 2005; Pandey et al. 2004). CREB mediates the activity of several signaling cascades, including cyclic-AMP–dependent protein kinase A, Ca^2+^/calmodulin-dependent protein kinases (CaMKs), and mitogen-activated protein kinase (Lonze and Ginty 2002; Pandey 2004). Studies from several laboratories have suggested a critical role for CREB in the regulation of alcohol’s effects and alcohol-related behaviors:

- Alcohol-induced damage to nerve cells (i.e., neurodegeneration) in the hippocampus was associated with decreased CREB functioning (Crews and Nixon 2009).
- Sensitivity to alcohol’s effects is associated with CREB transcriptional activity in another part of the brain called the cerebellum (Acquaah-Mensah et al. 2006). In addition, chronic ethanol exposure decreases CREB phosphorylation in the rat cerebellum (Yang et al. 1998).
- Abnormal CREB functioning in the amygdala—an important brain region implicated in regulating emotions—has been linked with a predisposition to anxiety^3^ and alcoholism (Pandey et al. 2004, 2005; Wand 2005). Amygdaloid pCREB and CBP levels both are significantly increased by acute ethanol exposure and decreased in rats undergoing withdrawal after chronic ethanol exposure (Pandey et al. 2008*a*,*b*). These results suggest that changes in CBP levels may be involved in the dynamic chromatin remodeling in the amygdala caused by acute and chronic ethanol exposure (see figures 4 and 5).

CREB signaling also helps regulate the expression of genes implicated in alcohol addiction, such as BDNF and neuropeptide Y (NPY) (Janak et al. 2006; Moonat et al. 2010; Pandey 2003; Valdez and Koob 2004). Lower NPY mRNA and protein levels have been found in the central (CeA) and medial nucleus of amygdala (MeA) of alcohol-preferring (P) rats compared with alcohol nonpreferring (NP) rats, suggesting a role for NPY in anxiety-like and alcohol-drinking behaviors (Pandey et al. 2005; Suzuki et al. 2004). NPY infusion into the CeA produced anxiety-reducing (i.e., anxiolytic) effects and decreased alcohol intake in P rats, most likely by leading to increased CaMK IV–dependent CREB phosphorylation in the CeA (Pandey et al. 2005; Zhang et al. 2010). Furthermore, P rats that had never been exposed to alcohol (i.e., were alcohol naïve) exhibited lower expression of other CREB target genes, such as BDNF and activity-regulated cytoskeleton-associated protein (Arc) in the CeA and MeA compared with NP rats (Moonat et al. 2011; Prakash et al. 2008). Finally, acute ethanol exposure increased CREB phosphorylation levels as well as the expression of BDNF and Arc in these amygdaloid structures of P, but not NP, rats (Moonat et al. 2011; Pandey et al. 2005).

Gamma-aminobutryric acid (GABA) is the main inhibitory brain signaling molecule (i.e., neurotransmitter), and alcohol is thought to produce many of its effects by facilitating GABA receptor functioning (Koob 2003*a*; Koob et al. 1998; Kumar et al. 2009). GABA activity in the amygdala is enhanced by exposure to alcohol and then decreased during alcohol withdrawal (Clapp et al. 2008; Koob 2003*a*). CREB seems to play a role in this process. A recent study showed that expression of one type of GABA receptors (i.e., the GABA_A_ receptor) was dependent on the formation of CREB heterodimers (Hu et al. 2008). Despite the tremendous amount of work that has been done on CREB, the epigenetic mechanisms for the regulation of gene expression via CREB and CBP interactions and its overall implications in alcoholism have not been well established and need to be investigated further.

#### Histone Deacetylases

HDACs are enzymes involved in covalent histone modifications that oppose the activity of HATs by inducing the removal of acetyl groups, thereby resulting in a condensed chromatin conformation and decreased gene expression levels in the cell (Grunstein 1997; Hsieh and Gage 2005; Turner 2002). Four distinct families of HDACs have been described, including class I (HDACs 1, 2, 3, and 8), class II (HDACs 4, 5, 6, 7, 9, and 10), class III (sirtuins), and class IV (HDAC 11). Class I HDACs mostly are found in the cell nucleus, whereas class II HDACs can be localized in the cytosol and/or nucleus. The class IV HDAC is located in the nucleus. Class I, II, and IV HDACs require zinc for proper functioning (i.e., are Zn^2+^ dependent). In contrast, class III HDACs require a molecule called nicotinamide adenosine dinucleotide (i.e., are NAD^+^ dependent) (Grayson et al. 2010; Kelly and Marks 2005; Lee et al. 2008).

Because of their epigenetic effects on chromatin structure and the role of epigenetic factors in the development of many tumors, HDAC inhibitors have been considered promising anticancer drugs for several years. Several of these agents currently are in clinical trials, and one of them, vorinostat (suberoylanilide hydroxamic acid), has been approved by the U.S. Food and Drug Administration for the treatment of a type of blood cancer (i.e, cutaneous T-cell lymphoma) (Dickinson et al. 2010; Dokmanovic and Marks 2005; Kelly and Marks 2005; Lee at al. 2008). Recent neuroscience research also advocates HDAC inhibitors as novel treatments for psychiatric and neurodegenerative disorders (Abel and Zukin 2008; Kanzantsev and Thompson 2008; Tsankova et al. 2007). For example, inhibitors of class I HDACs were able to reverse contextual memory deficits in a mouse model of AD; more specifically, HDAC 2 was involved in the negative regulation of learning and memory and related gene expression involved in synaptic plasticity (Guan et al. 2009; Kilgore et al. 2010). In addition, HDAC inhibitor treatment targeting HDAC 2 and HDAC 5 may have antidepressant effects (Covington et al. 2009; Tsankova et al. 2006). Finally, several studies assessing the modulatory effects of HDAC inhibition on cocaine abuse have indicated that these agents also may be beneficial in treating addictive processes related to drugs of abuse (Febo et al. 2009; Malvaez et al. 2010; Romieu et al. 2008; Wang et al. 2010).

Despite the abundance of research on the effects of HDAC inhibition on psychiatric disorders and drugs of abuse, the evaluation of HDAC inhibition in alcoholism research still is in its infancy. Emerging evidence from animal and human studies indicates that negative emotionality (e.g., anxiety or depression) is important in alcohol dependence, and sensitivity to anxiety may serve as a risk factor for AUDs (Cooper et al. 1995; Koob 2003*a*; Kushner et al. 1990; Pandey 2003). Alcohol has anxiolytic effects in humans, and rapid tolerance to these effects may stimulate alcohol drinking in alcoholics (Lipscomb et al. 1980; Moberg and Curtin 2009). Accordingly, rapid tolerance to alcohol’s anxiolytic effects may be crucial in the promotion of alcohol drinking and thereby contributes to the pathogenesis of alcohol dependence. In this context, recent discoveries involving the role of HDACs in alcohol tolerance and dependence are very promising. For example, studies have suggested that HDAC-induced chromatin remodeling in the amygdala may regulate the development of anxiety-like behaviors during ethanol withdrawal after chronic exposure and also may be paramount in rapid tolerance to the anxiolytic effects of ethanol in rats. The studies also found that HDAC activity was inhibited by acute ethanol in the amygdala of rats, and the expression of rapid tolerance to the anxiolytic effects of ethanol was reversed by treatment with a potent HDAC inhibitor, trichostatin A (TSA) (Pandey et al. 2008*a*; Sakharkar et al. 2012). Conversely, HDAC activity was increased in the amygdala of alcohol-withdrawn rats, which correlated with decreased histone acetylation and reduced NPY gene expression in the CeA and MeA of rats (Pandey et al. 2008*a*). Treatment with TSA during ethanol withdrawal prevented the development of anxiety-like behaviors and corrected the deficits in histone acetylation and NPY expression. These data suggest that epigenetic modifications involving HDACs result in a refinement of amygdaloid chromatin structure, which may be a contributing factor that alters the expression of genes implicated in alcohol tolerance and dependence (figures 4 and 5).

### Histone Methylation

Methylation of the chromatin (i.e., of both histones and DNA) is another important mechanism by which the cell regulates transcriptional activity (Holliday 2006). Histone methylation is mediated by histone methyltransferases (HMTs), whereas histone demethylation occurs via histone demethylases (HDMs) (Agger et al. 2008; Cheung and Lau 2005; Margueron et al. 2005; Shukla et al. 2008; Wysocka et al. 2006). Histone methylation is important in the regulation of memory formation (Gupta et al. 2010). Aberrant histone methylation at lysine residues has been implicated in several different psychiatric disorders (Akbarian and Huang 2009). For instance, mutations within genes encoding specific HMTs and HDMs have been associated with autism and mental retardation, respectively (Balemans et al. 2010; Tahiliani et al. 2007). In addition, chromatin remodeling at GABAergic gene promoters by a certain HMT may be involved in prefrontal cortex dysfunction during schizophrenia (Huang et al. 2007). Histone methylation, specifically involving histone H3, also has been linked with the expression levels for the receptor for the neurotransmitter glutamate, which has been implicated in schizophrenia because a hallmark of this disease is altered glutamate receptor functioning (Chavez-Noriega et al. 2002; Conn et al. 2009; Stadler et al. 2005). Tian and colleagues (2009) recently demonstrated that stimulation of a specific glutamate receptor (i.e., the *N*-methyl-d-aspartate [NMDA] receptor^4^) was associated with a decrease in histone H3 methylation and a concomitant increase in H3 acetylation at lysine 9 in rat hippocampal neurons. These findings also were linked with increased transcriptional activity of CREB and CBP at the BDNF gene promoter region 1. Taken together, these studies suggest that histone methylation may play an important role in the preservation of neuronal function by regulating expression of synaptic plasticity-associated genes.

## Epigenetic Modifications in Peripheral Tissues During Alcoholism

### Histone Acetylation and Methylation

Alcohol exposure not only affects the brain, leading to tolerance and dependence, but also can have serious harmful effects in other organs. For example, alcohol has deleterious effects on the liver that are associated with epigenetic changes (Shukla et al. 2008). Alcoholic liver disease is a debilitating condition associated with chronic alcohol abuse (Beier and McClain 2010). Studies in intact organisms (i.e., in vivo studies) have shown an increase in histone acetylation in the liver, lungs, spleen, and testes of rats acutely treated with ethanol (Kim and Shukla 2006). A study in isolated rat liver cells (i.e., primary rat hepatocytes) indicated that ethanol induced the acetylation of histone H3 at lysine 9 (Park et al. 2003). Also, by activating HAT, ethanol exposure caused increased histone acetylation (H3–lysine 9) in a promoter region of the gene encoding the alcohol-metabolizing enzyme alcohol dehydrogenase 1 (ADH 1) in rat hepatocytes (Park et al. 2005). In addition, exposure of hepatocytes to ethanol was associated with distinct histone H3 methylation patterns at lysine 9 and lysine 4, which correlated with increases and decreases, respectively, in gene expression (Pal-Bhadra et al. 2007).

Researchers recently demonstrated that another protein-modifying pathway (i.e., the ubiquitin–proteasome pathway^5^) is involved in epigenetic mechanisms regulating liver injury in alcoholic liver disease (Oliva et al. 2009). The investigators found that chronic ethanol feeding significantly inhibited the ubiquitin–proteasome pathway in rats, which was associated with an increase in histone acetylation and a decrease in histone methylation. Taken together, these data clearly suggest a promising epigenetic target for the treatment of ethanol-induced liver injury.

## DNA Methylation in the Brain and Periphery During Alcoholism

In addition to histone methylation, DNA methylation is an important epigenetic regulatory mechanism of gene expression that often can result in gene silencing (Comb and Goodman 1990; Feng and Fan 2009). DNA methylation is more specific than histone methylation and occurs in specific DNA sequences^6^ (Antequera 2003; Bestor 2000; Okano et al. 1999). DNA methylation within regulatory regions (i.e., promoters) of a gene has long been known to attenuate gene expression. More recently, however, DNA methylation within the gene also has been shown to regulate tissue- and cell-specific gene expression (Feng and Fan 2009; Maunakea et al. 2010).

DNA methylation patterns are established and maintained by enzymes called DNA methyltransferases (DNMTs); these patterns then are recognized by specific proteins (Robertson 2005; Sharma et al. 2010). Three main types of DNMTs regulate DNA methylation. For example, DNMT 1 maintains DNA methylation patterns, whereas DNMT 3a and DNMT 3b are involved in the de novo methylation of DNA (Okano et al. 1999). Similar to histones, DNA also undergoes removal of methyl groups (i.e., demethylation), and some enzymes involved in this process putatively have been identified (Bhattacharya et al. 1999; Metivier et al. 2008; Ooi and Bestor 2008).

DNMTs are abundantly expressed in postmitotic nerve cells (i.e., neurons) and are important for normal learning and memory (Feng et al. 2010). For example, an experimental approach in which animals learned to exhibit fear in a specific situation (i.e., contextual fear conditioning) was shown to increase the expression of DNMT 3a and 3b, but not DNMT 1 in the adult hippocampus, whereas infusion of a DNMT inhibitor blocked memory formation (Miller and Sweatt 2007). Furthermore, La Plant and colleagues (2010) found that DNMT3a expression was enhanced in a brain region called the nucleus accumbens by chronic cocaine use and chronic social stress, suggesting an important role for this enzyme in regulating emotional behavior and cocaine addiction.

DNA methylation also may play a role in psychiatric disorders, including alcohol and other drug dependence. For example, schizophrenia often is characterized by dysfunction of the GABA-using (i.e., GABAergic) signaling systems (Costa et al. 2003; Wassef et al. 2003). GABAergic neurons express high levels of DNMT 1, and excessive methylation (i.e., hypermethylation) of the promoter regions of genes involved in the GABA system may be a possible target for treating schizophrenia (Costa et al. 2007, 2009).

To date, no studies have assessed how DNMT enzymes are regulated during adaptive changes to ethanol exposure in the brain. Preliminary investigations from the author’s laboratory have suggested that acute ethanol exposure can significantly inhibit DNMT activity in the amygdala of rats. Other studies have shown that alcoholics have lower DNMT 3a and 3b expression in whole blood cells, with greater reductions found with higher blood ethanol concentrations (Bonsch et al. 2006). In addition, several other genes seem to be regulated through epigenetic mechanisms involving DNA methylation:

- The protein α-synuclein has been implicated in alcohol craving, and both the protein and mRNA levels of α-synuclein are elevated with chronic alcohol administration (Bonsch et al. 2005*a*; Walker and Grant 2006). Studies of human alcoholics found DNA hypermethylation of the promoter region for the α-synuclein gene in a type of white blood cells (i.e., peripheral mononuclear cells), which may be responsible for reduced craving under active alcohol-drinking conditions (Bonsch et al. 2005*b*; Bleich and Hillemacher 2009).
- Methylation also affects transcription of a gene encoding the endogenous opioid prodynorphin, a small protein (i.e., polypeptide) hormone involved in brain signaling that exists in several variants (i.e., alleles) that influence a person’s risk of alcoholism. A DNA variation (i.e., single nucleotide polymorphism) of this gene exists that overlaps with a CpG dinucleotide, which is subject to methylation. Methylation of this dinucleotide affects prodynorphin transcription in the prefrontal cortex of alcoholics (Taqi et al. 2011). Moreover, people who carried a nonrisk allele of the prodynorphin gene but in whom the CpG dinucleotide was methylated had a higher probability of developing alcohol dependence.
- Expression of the gene encoding a protein called homocysteine-induced endoplasmic reticulum protein (Herp) is lower in blood cells of alcoholics compared with healthy controls. This difference is related to promotor hypermethylation within the Herp gene in alcoholics (Bleich and Hillemacher 2009).
- The expression of the genes encoding the NMDA receptors, which as mentioned earlier are activated by the neurotransmitter glutamate, also seems to be regulated by DNA methylation. Thus, a clinical study demonstrated that in alcoholic patients the degree of methylation at the promoter region of the gene encoding the NMDA 2B receptor subtype (*NR2B*) during withdrawal negatively correlated with the severity of alcohol consumption (Biermann et al. 2009). Moreover, in primary cultured neurons, chronic intermittent ethanol exposure resulted in demethylation of a regulatory region of the *NR2B* gene, which correlated with increased *NR2B* gene expression (Qiang et al. 2010).

All of these findings suggest that ethanol-induced changes in DNA methylation may be an important factor in the regulation of gene expression that occurs during the complex processes of alcoholism pathogenesis.

DNA methylation represses gene expression via the actions of a protein called methyl CpG-binding protein-2 (MeCP2), which selectively binds to methylated DNA, thereby blocking transcription. Mutations in MeCP2 have been linked with a neurodevelopmental disorder, Rett syndrome (Bienvenu and Chelly 2006). Moreover, MeCP2 has been implicated in regulating behavioral responses to drugs of abuse (Feng and Nestler 2010). For example, changes in MeCP2 in the nucleus accumbens have been shown to contribute to the neural and behavioral responses to psychostimulants (Deng et al. 2010). MeCP2 also can control BDNF expression and cocaine intake (Im et al. 2010). Through its effects on BDNF expression, MeCP2 also may be important in regulating alcohol’s addictive properties (He et al. 2010), and BDNF expression is altered in various brain regions by acute and chronic ethanol exposure (Jeanblanc et al. 2009; Moonat et al. 2011; Pandey et al. 2008*b*).

### Role of DNA Methylation in Fetal Alcohol Spectrum Disorders

Fetal alcohol spectrum disorders (FASD) are developmental abnormalities related to the effects of in utero alcohol exposure on the developing fetus (Jones and Smith 1973; Jones et al. 1973). Several recent studies have emphasized how alcohol exposure can result in aberrant epigenetic regulatory mechanisms during development, leading to FASD. For example, alcohol consumption by the mother altered DNA methylation profiles in mouse embryos, resulting in neurofacial deficits and growth retardation, both of which are hallmarks of FASD (Liu et al. 2009). A recent study linked chronic alcohol use in men with lower-than-normal methylation (i.e., hypomethylation) of paternal sperm DNA, suggesting that genes from alcoholic males transferred through fertilization may result in offspring with FASD features (Ouko et al. 2009). Likewise, Weaver and colleagues (2004) demonstrated that maternal behavior was able to produce stable alterations in DNA methylation and chromatin structure in the hippocampus of their offspring that persisted into adulthood. However, this epigenetic maternal programming was reversible in adult offspring through methyl supplementation, suggesting that DNA methylation patterns that are formed early in life may not be permanent (Weaver et al. 2005). Finally, alcohol exposure seems to interfere with normal DNA methylation patterns of neural stem cell genes and to attenuate neural stem cell differentiation (Zhou et al. 2011). Taken together, these findings imply that epigenetic processes may play an important role in the mechanisms underlying FASD (Miranda 2011).

## miRNAs and Gene Expression in Alcoholism

In addition to mRNA, which is generated during gene expression and represents the coding sequences of the genes, several classes of noncoding RNA molecules exist that have regulatory functions. One of these classes is miRNA, which has been implicated in the regulation of gene expression and synaptic plasticity (Siegel et al. 2011). miRNAs control gene expression by interfering with the intricate processes of mRNA translation into a protein product and/or mRNA decay (Bartel 2009; Ghildiyal and Zamore 2009). Recent findings suggest that complex interactions occur between miRNAs and the epigenetic machinery (Bonasio et al. 2010). Likewise, other molecules known as large intergenic noncoding (linc) RNAs interact with chromatin-modifying complexes and regulate gene expression (Khalil et al. 2009).

Several studies have implicated miRNAs in the cellular effects of ethanol use and abuse (Miranda 2010; Pietrzykowski 2010). For example, miRNA-9 (miR-9) posttranscriptionally regulates big potassium (BK) channel mRNA variants that encode different types of BK channels with different sensitivities to alcohol (Pietrzykowski et al. 2008). Acute ethanol exposure increased the expression of miR-9 in neurons isolated from certain regions of the adult rodent brain (i.e., the supraoptic nucleus and striatum), which correlated with reduced BK channel expression (Pietrzykowski et al. 2008). Alcohol-related BK channel dysfunction may play an important role in the development of alcohol tolerance (Cowmeadow et al. 2005; Pietrzykowski 2008). On the other hand, physiologically relevant ethanol concentrations, similar to those attained by alcoholics, can significantly suppress the expression of four other miRNAs (i.e., miR-21, miR-335, miR-153, and miR-9) (Sathyan et al. 2007). Together, these studies highlight the emerging role of miRNAs as mediators of epigenetic modifications that may be involved in ethanol’s actions.

## Conclusions

**Figure 1:**
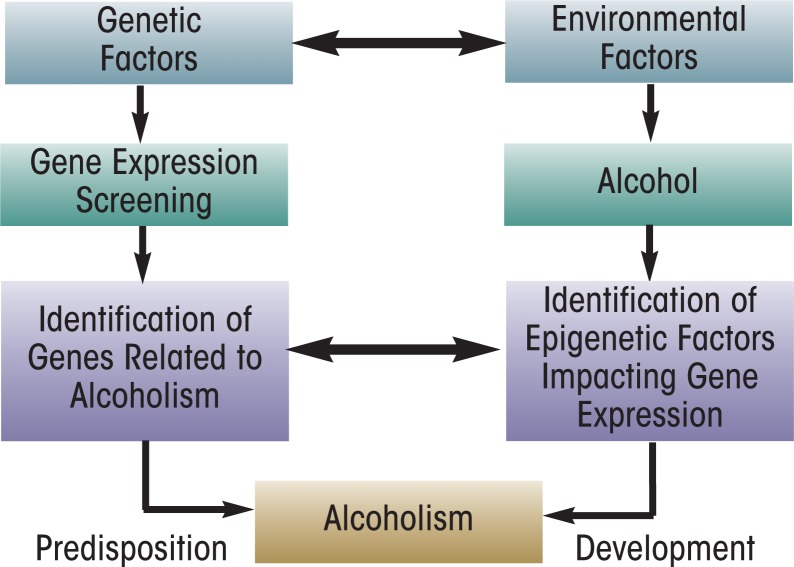
A hypothetical model for the interactions of genetic and environmental factors in the predisposition to and development of alcoholism. Whole genome expression profiling in human and animal models has identified several potential candidate genes that may be associated with increased risk for alcoholism and alcohol abuse. Emerging evidence suggests that genomic function can be modulated by alcohol-induced epigenetic modification of histones (acetylation and methylation) and DNA methylation, which can lead to the development of alcoholism. Both genetic and epigenetic factors may interact in the underlying mechanisms of alcoholism.

**Figure 2:**
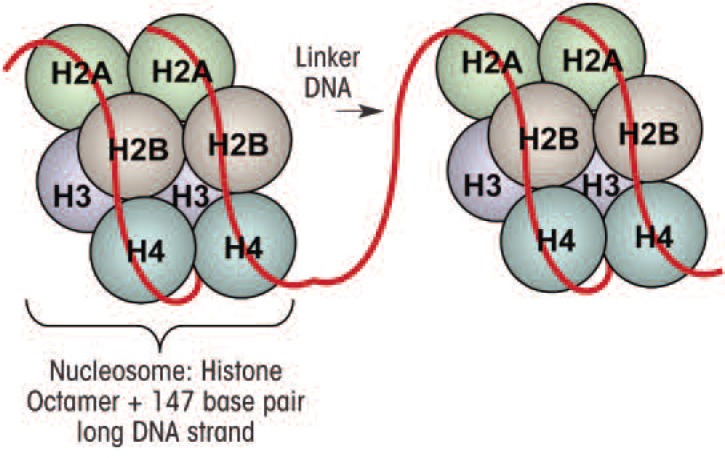
Schematic representation of units of chromatin known as nucleosomes. Each nucleosome is comprised of 147 base pairs of DNA wrapped around the histone octamer made up of a heterotetramer of histones H3 and H4 and two heterodimers of H2A and H2B. Two nucleosomes are connected by the linker DNA.

**Figure 3:**
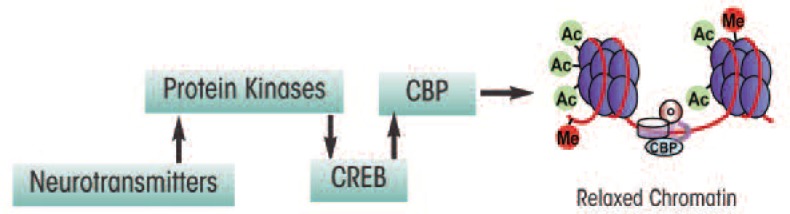
A schematic representation of the signaling pathway showing the possible role for cyclic-AMP responsive element binding (CREB) protein and CREB-binding protein (CBP) in the epigenetic regulation of gene expression. CREB is activated after its phosphorylation (pCREB) by different protein kinases, which further recruits CBP to bind to the DNA. CBP also has intrinsic histone acetyltransferase (HAT) activity by which it increases histone aceylation and relaxes the chromatin, which in turn makes the DNA more accessible to the transcriptional machinery and thereby facilitates gene transcription.

**Figure 4:**
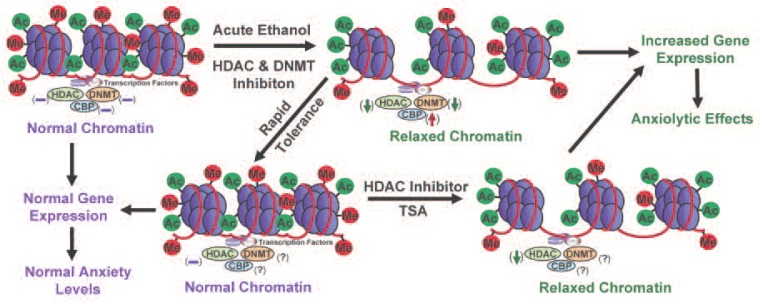
A schematic diagram depicting possible epigenetic mechanisms acting in neuronal circuits of the amygdala that may contribute to rapid tolerance to the anxiolytic effects of ethanol. Acute ethanol exposure can inhibit both histone deacetylase (HDAC) and DNA methyltransferase (DNMT) activities (Pandey et al. 2008*a*). This inhibition correlates with increased amygdaloid levels of CREB-binding protein (CBP), which has histone acetyltransferase (HAT) activity. The observed changes in HDAC activity and CBP levels also correlate with increased histone acetylation (H3-K9 and H4-K8) in the central and medial nucleus of amygdala, resulting in a relaxed chromatin structure. As a result, the transcriptional machinery can more easily access the DNA, leading to increased gene expression. Increased amygdaloid expression of certain genes, such as neuropeptide Y or brain-derived neurotrophic factor, may mediate the anxiolytic effects of acute ethanol exposure (Pandey et al. 2008*a*, *b*). Cellular tolerance at the level of HDAC-induced chromatin remodeling in the amygdala may be operative in rapid tolerance to the anxiolytic effects of ethanol (Sakharkar et al. 2012). NOTE: (↓) = decrease; (↑) = increase; (−) = normal; (?) = unknown; Me = methylation site; Ac = acetylation site.

**Figure 5:**
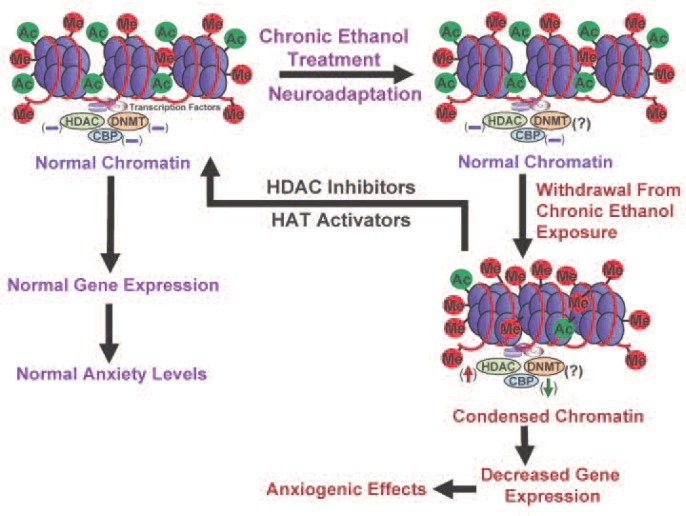
A representative model of possible epigenetic mechanisms acting in neuronal circuits of the amygdala that may contribute to the development of anxiety-like behaviors during ethanol withdrawal after chronic exposure in rats. Chronic ethanol exposure and the concomitant neuroadaptations in the amygdala of rats do not significantly affect levels of histone acetylation, CREB-binding protein (CBP), or histone deacetylase (HDAC) activity, because these were altered by acute ethanol exposure (see figure 4). However, withdrawal after chronic ethanol exposure is associated with increased HDAC activity and decreased levels of CBP and associated histone acetylation (Pandey et al. 2008*a*). As a result, the chromatin configuration may become more condensed, which limits accessibility of the transcriptional machinery to the DNA. This may result in decreased gene expression levels of neuropeptide Y and brain-derived neurotrophic factor, both of which have been linked to increased anxiety-like behaviors (i.e., have anxiogenic effects) following withdrawal from chronic ethanol exposure (Pandey et al. 2008*a*,*b*). HDACs and histone acetyltransferases (HATs) are promising targets in the possible reversal of these anxiogenic consequences of withdrawal. Thus, pharmacological treatment using potent HDAC inhibitors or HAT activators may lead to the normalization of reduced histone acetylation and subsequent stabilization of gene expression and anxiety levels. NOTE: (↓) = decrease; (↑) = increase; (−) = normal; (?) = unknown; Me = methylation site; ac = acetylation site

Epigenetics is an emerging area of research in the neuroscience field. The work done so far indicates that chromatin remodeling resulting from histone modifications in the amygdala may be important in the effects of both acute and chronic ethanol exposure (see figures 4 and 5). Other evidence has identified DNA methylation as a critical regulator of gene expression levels, which also may play a critical role in alcoholism. However, the specific effects of different types of HDAC in alcohol’s action or the regulation of various DNMTs in the brain by alcohol exposure still remain unclear. It also would be interesting to determine the role of other important enzymes involved in remodeling the chromatin structure, such as DNA and histone demethylases and HMTs, in alcohol’s effects. Currently, the alcohol-related regulation of histone and DNA methylation in the brain and other organ systems are not well understood. Future studies will provide a more comprehensive picture of epigenetic mechanisms regulated by alcohol in order to increase our understanding of overall genomic function in alcoholism (figure 1). Nonetheless, these early research findings clearly provide evidence that compounds that inhibit HDACs may be promising future therapeutic agents in the treatment of alcoholism (figures 4 and 5).

## Footnotes

1 Gene expression is the process of converting the genetic information encoded in the DNA into actual gene products (i.e., proteins). This process involves two steps. In the first step, called transcription, the genetic information comprising one gene is copied into an intermediary molecule called messenger RNA (mRNA). After the mRNA is processed further using a variety of posttranscriptional modifications, it is used as a template to generate proteins in a process called translation.

2 Heterotetramers consist of four subunits that are not all identical, and heterodimers consist of two subunits that are not identical.

3 Anxiety is an early withdrawal symptom that plays a major role in relapse, helping to maintain a dependent state in certain alcoholics (Cooper et al. 1995; Koob 2003a; Kushner et al. 1990; Menzaghi et al. 1994).

4 NMDA receptors mediate excitatory neurotransmission in the brain. These ubiquitous receptors play a role in the development of neurons and synaptic plasticity (Wheal et al. 1998). Modulation of NMDA receptor function may mediate the development of tolerance to alcohol as well as alcohol withdrawal symptoms (Kumari and Ticku 2000).

5 The ubiquitin–proteasome pathway is a series of reactions by which unwanted or defective proteins are marked for degradation by the addition of ubiquitin molecules and subsequently destroyed by large protein complexes known as proteasomes. Accurate functioning of this pathway is essential for many cellular processes, the cell cycle, and regulation of gene expression.

6 Specifically, DNA methlylation occur at the 5’-position of cytosines found in cytosine–guanosine (CpG) dinucleotides within CpG-rich DNA regions (i.e., CpG islands).
